# Crystal structures and Hirshfeld surfaces of four meth­oxy­benzaldehyde oxime derivatives, 2-MeO-*X*C_6_H_3_C=NOH (*X* = H and 2-, 3- and 4-MeO): different conformations and hydrogen-bonding patterns

**DOI:** 10.1107/S2056989018014020

**Published:** 2018-10-09

**Authors:** Ligia R. Gomes, Marcus V. N. de Souza, Cristiane F. Da Costa, James L. Wardell, John Nicolson Low

**Affiliations:** a REQUIMTE, Departamento de Química e Bioquímica, Faculdade de Ciências da Universidade do Porto, Rua do Campo Alegre, 687, P-4169-007, Porto, Portugal; b FP-ENAS-Faculdade de Ciências de Saúde, Escola Superior de Saúde da UFP, Universidade Fernando Pessoa, Rua Carlos da Maia, 296, P-4200-150 Porto, Portugal; c Instituto de Tecnologia em Fármacos e Farmanguinhos, Fundação Oswaldo Cruz, 21041-250 Rio de Janeiro, RJ, Brazil; d Department of Chemistry, University of Aberdeen, Meston Walk, Old Aberdeen, AB24 3UE, Scotland

**Keywords:** crystal structure, oxime derivative, hydrogen bonding

## Abstract

The crystal structures of four (*E*)-meth­oxy­benzaldehyde oxime derivatives, namely (2-meth­oxy­benzaldehyde oxime, **1**, 2,3-di­meth­oxy­benzaldehyde oxime, **2**, 4-di­meth­oxy­benzaldehyde oxime, **3**, and 2,5-di­meth­oxy­benzaldehyde oxime, **4**, are discussed. The arrangements of the 2-meth­oxy group and the H atom of the oxime unit are *s-cis* in compounds **1**–**3**, but in both independent mol­ecules of compound **4**, the arrangements are *s-trans*. There is also a difference in the conformation of the two mol­ecules in **4**, involving the orientations of the 2- and 5-meth­oxy groups. The primary inter­molecular O—H(oxime)⋯O(hy­droxy) hydrogen bonds generate *C*(3) chains in **1** and **2**. In contrast, in compound **3**, the O—H(oxime)⋯O(hy­droxy) hydrogen bonds generate symmetric *R*_2_^2^(6) dimers. A more complex dimer is generated in **4** from the O—H(oxime)⋯O(hy­droxy) and C—H(2-meth­oxy)⋯O(hy­droxy) hydrogen bonds. In all cases, further inter­actions, C—H⋯O and C—H⋯π or π–π, generate three-dimensional arrays. Hirshfeld surface and fingerprint analyses are discussed.

## Abstract 2

The crystal structures of four (*E*) -meth­oxy­benzaldehyde oxime derivatives, namely (2-meth­oxy­benzaldehyde oxime, **1**, 2,3-di­meth­oxy­benzaldehyde oxime, **2**, 4-di­meth­oxy­benzaldehyde oxime, **3**, and 2,5-di­meth­oxy­benzaldehyde oxime, **4**, are discussed. The arrangements of the 2-meth­oxy group and the H atom of the oxime unit are *s-cis* in compounds **1**–**3**, but in both independent mol­ecules of compound **4**, the arrangements are *s-trans*. The primary inter­molecular O—H(oxime)⋯O(hy­droxy) hydrogen bonds generate *C*(3) chains in **1** and **2**. In contrast, in compound **3**, the O—H(oxime)⋯O(hy­droxy) hydrogen bonds generate symmetric 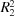
(6) dimers. A more complex dimer is generated in **4** from the O—H(oxime)⋯O(hy­droxy) and C—H(2-meth­oxy)⋯O(hy­droxy) hydrogen bonds.

## Chemical context   

In the plant kingdom, oximes play a vital role in metabolism (Sørensen *et al.*, 2018 ▸). Aldoximes, *R*CH=NOH, are found in many biologically active compounds (Abele *et al.*, 2008 ▸; Nikitjuka & Jirgensons, 2014 ▸), having a diverse range of uses including as anti-tumour agents (Martínez-Pascual *et al.*, 2017 ▸; Qin *et al.*, 2017 ▸; Canario *et al.*, 2018 ▸; Huang *et al.*, 2018 ▸), acaricidal and insecticidal agents (Dai *et al.*, 2017 ▸), thymidine phospho­rylase inhibitors (Zhao *et al.*, 2018 ▸), anti-microbial agents (Yadav *et al.*, 2017 ▸), bacteriocides (Kozlowska *et al.*, 2017 ▸), anti-inflammatory agents (Mohassab *et al.* 2017 ▸), and in the treatment of nerve-gas poisoning (Lorke *et al.*, 2008 ▸; Voicu *et al.*, 2010 ▸; Katalinić *et al.*, 2017 ▸; Radić *et al.*, 2013 ▸).

**Figure 1:**
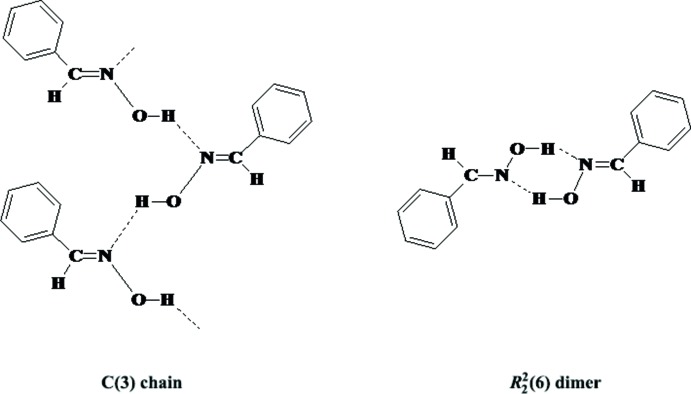
Illustrations of the *C*(3) chains and 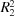
(6) dimers formed by oximes

Benzaldehyde oximes, ArCH=NOH, with their –CH=N—OH functional group are ideally arranged for classical O—H⋯O and/or O—H⋯N hydrogen bonding. The last survey of the classical hydrogen-bonding patterns in benzaldehyde oximes reported in 2010 (Low *et al.*, 2010 ▸) confirmed that the most frequently found arrangements, with the exception of salicylaldoxines, are 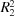
(6) dimers and *C*(3) chains, Fig. 1 ▸. Aakeröy *et al.* (2013 ▸) reported the percentages of 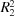
(6) dimers and *C*(3) chains found in non-salicylaldoxine to be *ca* 72 and 24%, respectively – similar percentages can be derived from a recent survey of the Cambridge Structural Database (CSD Version 5.39, August 2018 update; Groom *et al.*, 2016 ▸). Hydrogen bonds are considered to be the strongest and most directional of inter­molecular inter­actions in mol­ecules (Etter, 1990 ▸) and thus play the major roles in determining the overall supra­molecular structures. However, the involvement of weaker inter­molecular inter­actions, such as C—H⋯O hydrogen bonds, π–π inter­actions and inter­actions involving the substituents, can have a significant influence on the supra­molecular arrays generated. In a continuation of recent studies on aldoximes (Low *et al.* 2018 ▸; Gomes *et al.*, 2018 ▸), we have determined the crystal structures of four meth­oxy­benzaldehyde derivatives, namely 2-MeO-*X*-C_6_H_3_CH=NOH where *X* = H in **1**, *X* = 3-MeO in **2**, *X* = 4-MeO in **3** and *X* = 5-MeO in **4**. The aim of the study was to further investigate the occurrence of 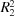
(6) dimers and *C*(3) chains in a series of related compounds.
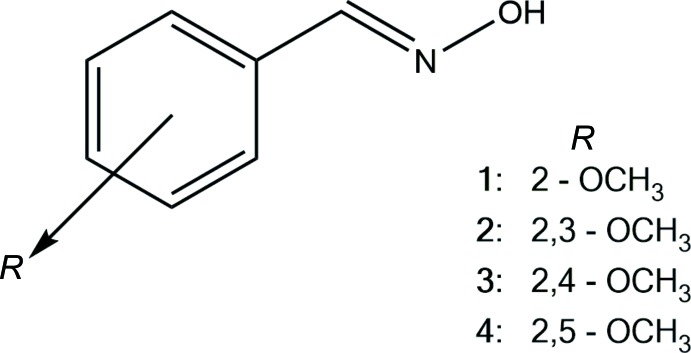


## Structural commentary   

**Figure 2:**
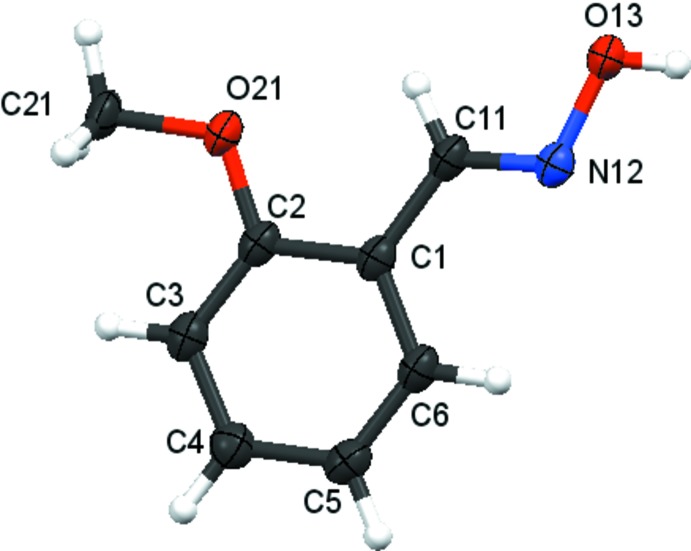
Atom arrangements and numbering scheme for compound **1**. Displacement ellipsoids are drawn at the 50% probability level.

**Figure 3:**
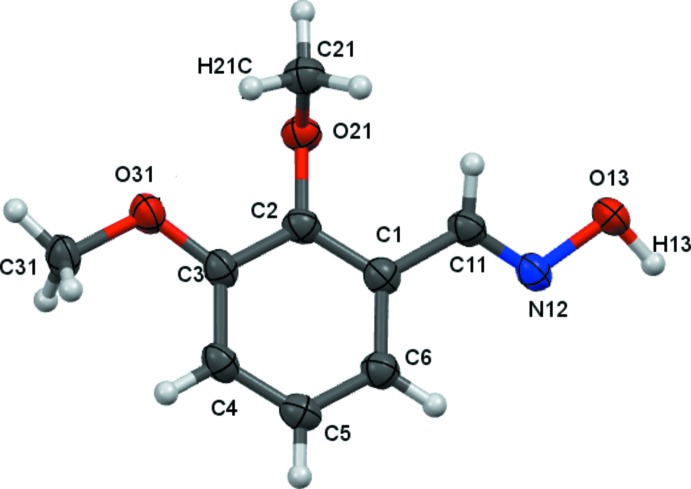
Atom arrangements and numbering system for compound **2**. Displacement ellipsoids are drawn at the 50% probability level.

**Figure 4:**
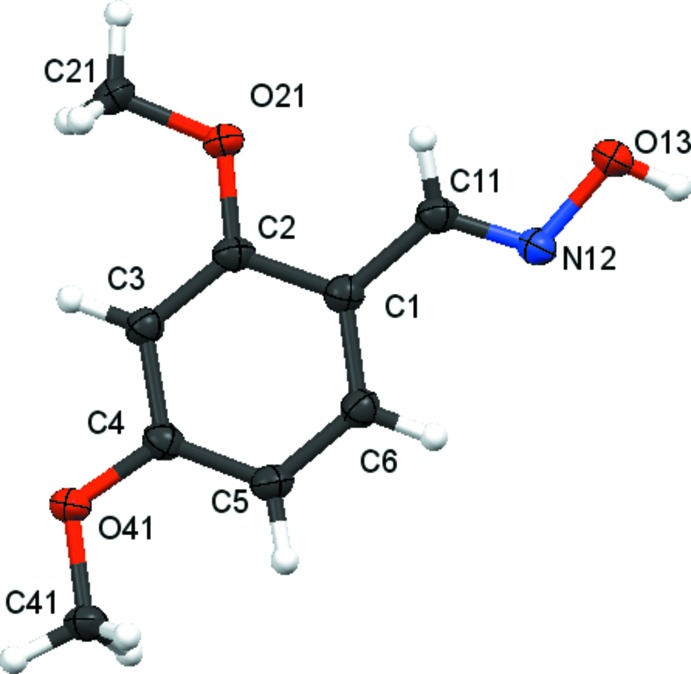
Atom arrangements and numbering system for compound **3**. Displacement ellipsoids are drawn at the 50% probability level.

**Figure 5:**
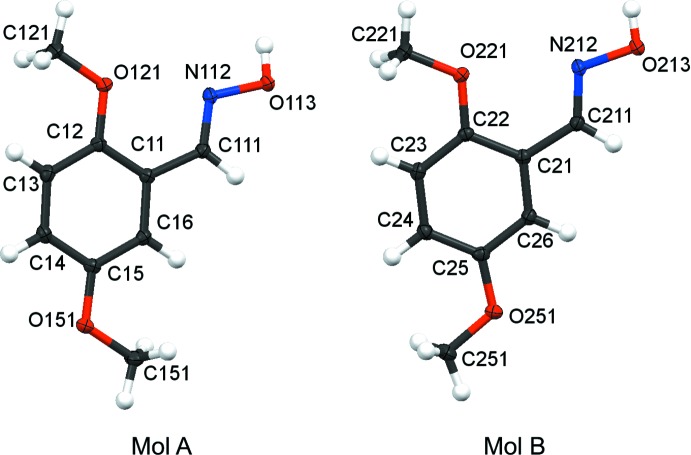
Atom arrangements and numbering system for the two independent mol­ecules, Mol A and Mol B, of compound **4**. Displacement ellipsoids are drawn at the 50% probability level.

There are no unusual features in the mol­ecular structures. Compound **1** crystallizes in the *ortho­rhom­bic* space group *Pna*2_1_ with one mol­ecule in the asymmetric unit (Fig. 2 ▸), compound **2** crystallizes in the *ortho­rhom­bic* space group *P*2_1_2_1_2_1_ with one mol­ecule in the asymmetric unit (Fig. 3 ▸), compound **3** crystallizes in the *triclinic* space group *P* 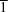 with one mol­ecule in the asymmetric unit (Fig. 4 ▸), and compound **4** crystallizes in the *monoclinic* space group, *P*2_1_/c with two independent mol­ecules, Mol A and Mol B, in the asymmetric unit (Fig. 5 ▸). The geometry about the oxime moiety in all mol­ecules is (*E*). In compounds **1**–**3**, the 2-meth­oxy group and the hydrogen of the oxime moiety have an *s-cis* arrangement. In contrast, in both mol­ecules of compound **4**, the 2-meth­oxy group and the hydrogen atom of the oxime moiety have an *s-trans* arrangement. The *s-trans* arrangement of the 2-alk­oxy group and hydrogen atom of the oxime units in compound **4** is very much rarer than the *s-cis* arrangement found in compounds **1**–**3** and other non-salicylaldoximes. A search of the Cambridge Structural Database (CSD Version 5.39, August 2018 update; Groom *et al.*, 2016 ▸) revealed that only salicyl­aldoximes and 2-alk­oxy­benzaldehyde oxime (*E*)-2-({2-[(*E*)-(hy­droxy­imino)­meth­yl]phen­oxy}meth­yl)-3-*p*-tolyl­acryl­o­nitrile (LAQRIG; Suresh *et al.* 2012 ▸) had this *s-trans* arrangement. In contrast, the isomer 2-({2-[(hy­droxy­imino)meth­yl]phen­oxy}meth­yl)-3-(2-methyl­phen­yl)acrylo­nitrile (GARNEU; Govindan *et al.*, 2012*a* ▸) and some similar compounds such as (*E*)-2-({2-[(*E*)-(hy­droxy­imino)­meth­yl]phen­oxy}meth­yl)-3-phenyl­acrylo­nitrile (LAQRUS; Govindan *et al.*, 2012*b* ▸) had the *s*-*cis* arrangement.

There is a conformational difference between the two independent mol­ecules Mol A and Mol B of compound **4**. This difference is in the orientation of the two meth­oxy groups, see Fig. 5 ▸: in Mol A the orientation is *s-trans* and in Mol B, it is *s-cis*. As expected for a 1,2,3-tris­ubstituted benzene derivative, compound **4** is the least planar of the four oxime derivatives, with the 2-meth­oxy substituent furthest out of the plane of the attached phenyl group, see Table 1 ▸.

## Supra­molecular features   

### Hydrogen bonding   

**Figure 6:**
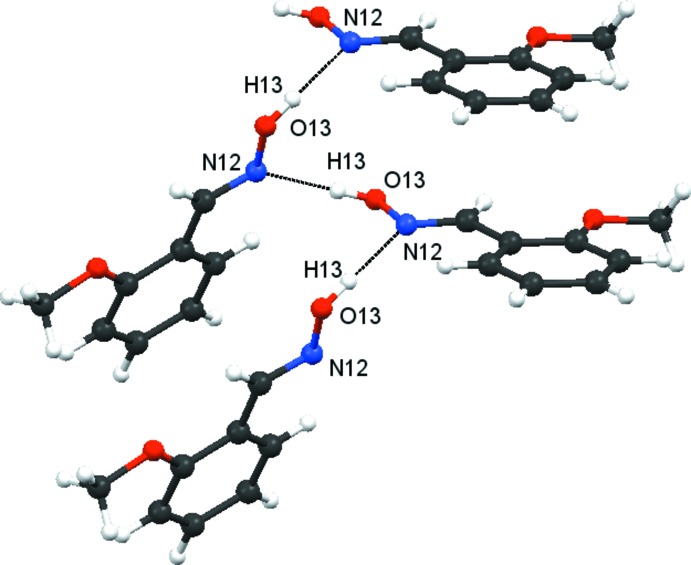
Compound **1**. Part of a *C*(3) chain formed by O13—H13⋯·N12 hydrogen bonds (dashed lines; see Table 2 ▸).

**Figure 7:**
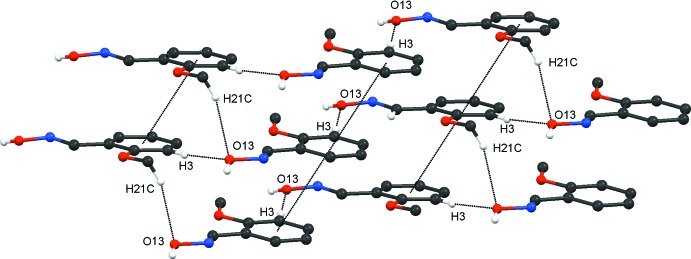
Compound **1**. Part of the arrangement generated from the combination of hydrogen bonds and π–π inter­actions (dashed lines; see Table 2 ▸).

In the crystal of **1**, mol­ecules are primarily linked by strong O13—H13⋯N12^i^ hydrogen bonds (Table 2 ▸), forming *C*(3) chains, illustrated in Fig. 6 ▸. Also present in compound **1** are two weaker hydrogen bonds, namely, C3—H3⋯O13^ii^ and C21—H21*C*⋯O13^iii^, as well as a weak π–π stacking inter­action [*Cg*⋯*Cg* ^iv^ = 4.025 (2) Å: slippage 2.105 Å: symmetry code; *x*, *y*, *z* − 1]. These three inter­actions generate the mol­ecular arrangement shown in Fig. 7 ▸. The C3—H3⋯O13^ii^ hydrogen bonds generate *C*7 chains in the *c*-axis direction, while the C21—H21*C*⋯O13^iii^ hydrogen bonds form *C*(8) spiral chains along the *a*-axis direction: together these hydrogen bonds form 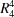
(22) rings. The tilted π–π stacks propagate in the *c*-axis direction. The involvement of the weaker C3—H3⋯O13^ii^, C21—H21*C*⋯O13^iii^ and π–π inter­actions, along with the stronger O13—H13 ⋯N12^i^ hydrogen bonds, creates the three-dimensional structure for **1**.

**Figure 8:**
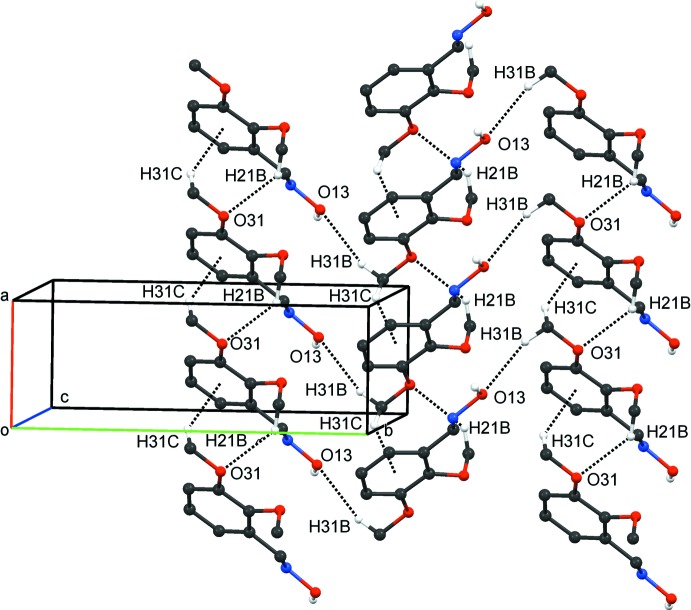
Compound **2**. Part of the arrangement generated form C21—H21*B*⋯O31, C31—H31*B*⋯O13 and π–π inter­actions (dashed lines; see Table 3 ▸).

As in **1**, mol­ecules of **2** are primarily linked by strong O13—H13 ⋯N12^i^ hydrogen bonds (Table 3 ▸), forming *C*(3) chains: as such chains are very similar to those in compound **1**, see Fig. 6 ▸, an illustration has not been provided for the *C*(3) chain in compound **2**. Other inter­molecular inter­actions in **2** are the weaker C21—H21*B*⋯O31^iii^ and C31—H31*B*⋯O13^iv^ hydrogen bonds and a C31—H31*C*⋯*Cg*1^v^ inter­action involving the C1–C6 ring. These three inter­actions combine to form the arrangement illustrated in Fig. 8 ▸. The C21—H21*B*⋯O31^iii^ hydrogen bonds on their own generate *C*(6) chains, which propagate in the *a*-axis direction while the C31—H31*B*⋯O13^iv^ hydrogen bonds generate spiral *C*(9) chains in the *b*-axis direction. Together these hydrogen bonds generate a network of 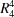
(26) rings. The C31—H31*C*⋯*Cg*1^v^ inter­actions lead to chains along the *a-*axis direction. The involvement of the weaker C21—H21*B*⋯O31^iii^, C31—H31*B*⋯O13^iv^ C and C—H⋯π inter­actions, along with the stronger O13—H13 ⋯N12^i^ hydrogen bonds, creates a three-dimensional structure for **2**. C4—H4⋯O12^ii^ hydrogen bonds also occur.

**Figure 9:**
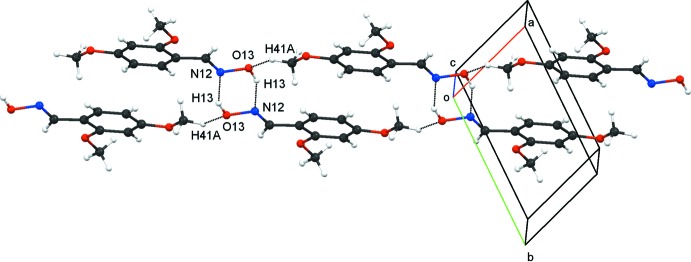
Compound **3**. A two-mol­ecule-wide ribbon generated from linking the 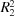
(6) dimers, formed by pairs of strong O13—H13—N12 hydrogen bonds and by weaker C41—H41*A*⋯O13 hydrogen bonds (dashed lines; see Table 4 ▸).

In compound **3**, 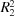
(6) dimers are generated from strong O13—H13⋯N12^i^ hydrogen bonds (Table 4 ▸), as illustrated in Fig. 9 ▸. Linkages of these 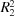
(6) dimers by weaker C41—H41*A*(meth­oxy)⋯O13^ii^ hydrogen bonds provide a two-mol­ecule-wide ribbon. Within the ribbons are 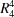 (22) rings as well as the 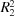
(6) rings. An additional inter­action in **3** is the C41—H41*C*⋯*Cg*1^iii^ inter­action, which generates a tilted ladder assembly, propagating in the *a*-axis direction, with the 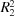
(6) rings acting as the rungs and the C41—H41*C*⋯*Cg*1^iii^ inter­actions as the supports.

**Figure 10:**
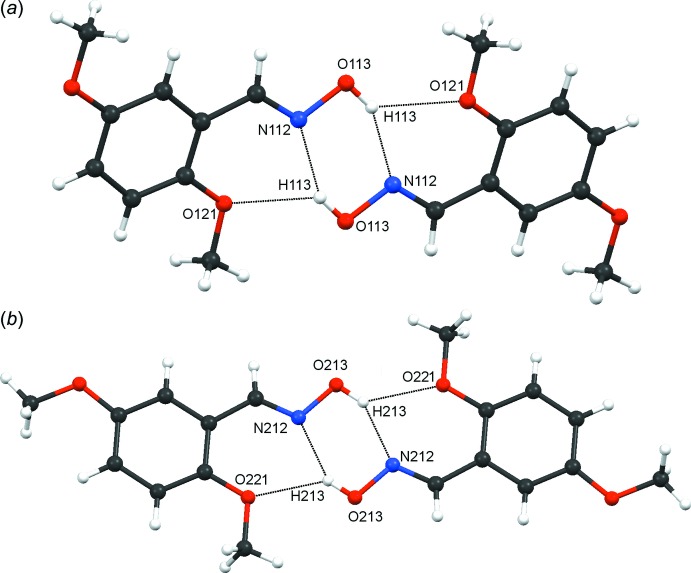
Compound **4**. Symmetric dimers of (*a*) Mol A and (*b*) Mol B. Hydrogen bonds (see Table 5 ▸) are shown as dashed lines.

In compound **4**, each of the two independent mol­ecules forms symmetric dimers, see Fig. 10 ▸. These are generated from combinations of O113—H113⋯N112^i^ and O113—H113⋯O121^i^ hydrogen bonds (Table 5 ▸) for Mol A and O213—H213⋯N212^ii^ and O213—H213⋯O221^ii^ hydrogen bonds for Mol B. In each case, the dimers contain three rings, two 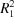
(6) and one 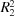
(6). There are short N⋯N distances across the 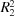
(6) dimer rings, 2.8595 (12) Å for MolA and 2.8956 (12) Å for Mol B, each being less than the sum of the van der Waals radius (3.10 Å) for two N atoms.

**Figure 11:**
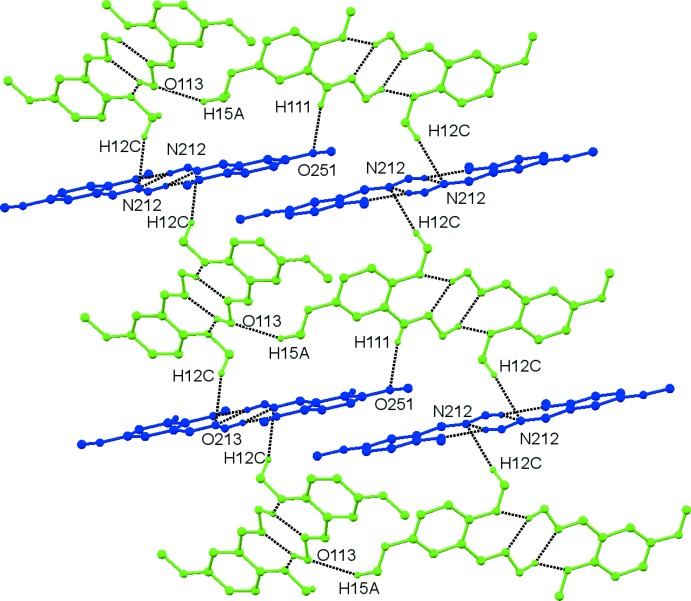
Compound **4**. Symmetric dimers of Mol A (green) and Mol B (blue). Inter­molecular inter­actions (see Table 5 ▸) are shown as dashed lines.

The links between the two different dimers of **4** are provided by a number of C—H⋯O and C—H⋯π inter­actions, listed in Table 5 ▸. Fig. 11 ▸ restricts the contacts to just the C—H⋯O hydrogen bonds, namely C121—H12*C*⋯N212^iii^, C111—H111⋯O251 and C151—H15*A*⋯O113^iv^. To facilitate the viewing of the connection in Fig. 11 ▸, the two different dimers are drawn in different colours.

### Hirshfeld surface analysis   

**Figure 12:**
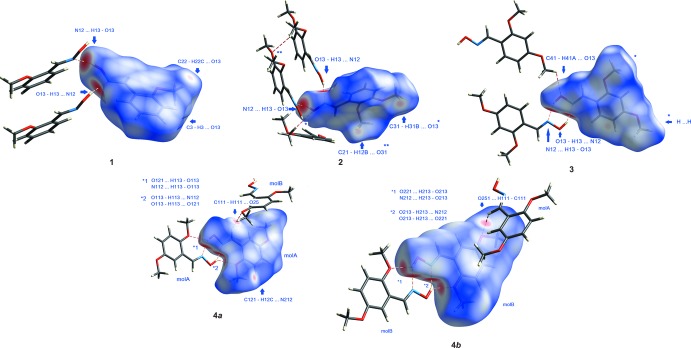
Hirshfeld surfaces for compounds **1**–**4**. In each case, the inter­actions related to the red areas are designated.

**Figure 13:**
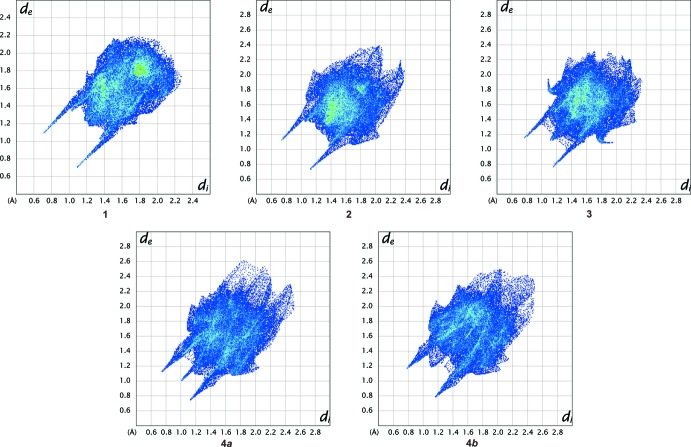
Fingerprint plots for compounds **1**–**4**.

Hirshfeld surfaces (Spackman & Jayatilaka, 2009 ▸) and two-dimensional fingerprint (FP) plots (Spackman & McKinnon, 2002 ▸), provide complementary information concerning the inter­molecular inter­actions discussed above. The analyses were generated using *Crystal Explorer3.1* (Wolff *et al.*, 2012 ▸). The Hirshfeld surfaces mapped over *d*_norm_ for **1**–**4** are illus­trated in Fig. 12 ▸. The red areas on the surfaces correspond to close contacts. The fingerprint plots are shown in Fig. 13 ▸. In all of the FP plots, the pair of spikes pointing south-west relate to the N—H contacts, which in compounds **1** and **2** are involved in the *C*(3) chains, while in compounds **3** and **4**, they are responsible for the creation of the dimers. In compound **3**, the fins ending at *d*_e_, *d*_i_ = 1.9,1.1 Å are due to C(π)⋯H/C(π)⋯H contacts. The FP plots for Mol A and Mol B of compound **4** are asymmetric because of the different inter­actions of each mol­ecule. The double wings in the FP plot for Mol A in the second quadrant are complementary to those displayed in the fourth quadrant by MolB and relate to C⋯H close contacts connecting the two mol­ecules. The spike ending at *d*_i_, *d*_e_ = 1.1 Å in Mol A is due to H⋯H contacts.

The percentages of the various atom–atom contacts, derived from the fingerprint plots, for the four compounds are shown in Table 6 ▸. The fact that compound **1** has only one meth­oxy group while the isomers, **2**–**4**, have two is reflected in the greater percentages of contacts involving the oxygen close contacts. The *C*(3)-chain-forming compounds **1** and **2** show higher percentages of H⋯H and C⋯C contacts, but a lower percentage of H⋯C/C⋯H contacts, than the dimer-forming compounds **3** and **4.**

## Database survey   

A search of the Cambridge Structural Database survey (CSD Version 5.39, August 2018 update; Groom *et al.*, 2016 ▸) revealed compounds similar to **2** and **3.** The classical hydrogen bonds in 3,5-di­meth­oxy­benzene oxime generate *C*(3) chains (VUZJAC; Dong *et al.*, 2010 ▸). No benzene oxime derivative with only meth­oxy substituents has been reported in the database to form an 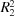
(6) or related dimer. The structure has been reported of 3,4,5-tri­meth­oxy­benzene oxime (MEQDAO; Chang, 2006 ▸) in which classical hydrogen bonds, formed between the oxime unit and the 4- and 5-meth­oxy moieties, but not the 2-meth­oxy group, result in the formation of a tetra­mer. The water mol­ecule in 3,4.5-tri­meth­oxy­benzene monohydrate (HESWUY; Priya *et al.*, 2006 ▸) is strongly involved in the hydrogen-bonding arrangements.

There are 376 structures, (411 fragments) in the CSD database with oxime 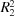
(6) dimers in which the N⋯N distance across the ring is less than or equal to 3.10 Å, the sum of two N-atom van der Waals radii. The H⋯O hydrogen-bond distance range was restricted to 1.739–2.285 Å to exclude improbable O⋯H distances based on a statistical analysis in *Mercury* (Macrae *et al.*, 2006 ▸). The N⋯N distances range from 2.727 to 3.097 Å with a mean value of 2.987 Å. There are 27 structures within the range 2.838 to 2.909 Å in which our values of 2.8595 (12) Å for MolA and 2.8956 (12) Å for MolB of compound**4** lie. Only single-crystal organic compounds were searched for with no limit on the *R* factor.

## Synthesis and crystallization   

The title compounds were prepared from hy­droxy­amine and the corresponding benzaldehyde in methanol in the presence of potassium carbonate and were recrystallized from methanol solutions, m.p. = 364–365 K for compound **1**, 371–373 K for **2**, 378–380 K for **3** and 370–371 K for **4**.

## Refinement details   

Crystal data, data collection and structure refinement details are summarized in Table 7 ▸. All hy­droxy hydrogen atoms were refined isotropically. C-bound H atoms were refined as riding with C—H = 0.95–0.98Å and *U*_iso_(H) = 1.2–1.5*U*_eq_(C).

## Supplementary Material

Crystal structure: contains datablock(s) 1, 2, 3, 4, global. DOI: 10.1107/S2056989018014020/qm2129sup1.cif

Structure factors: contains datablock(s) 1. DOI: 10.1107/S2056989018014020/qm21291sup2.hkl

Structure factors: contains datablock(s) 2. DOI: 10.1107/S2056989018014020/qm21292sup3.hkl

Structure factors: contains datablock(s) 3. DOI: 10.1107/S2056989018014020/qm21293sup4.hkl

Structure factors: contains datablock(s) 4. DOI: 10.1107/S2056989018014020/qm21294sup5.hkl

Supporting information file. DOI: 10.1107/S2056989018014020/qm21291sup6.cml

Supporting information file. DOI: 10.1107/S2056989018014020/qm21292sup7.cml

Supporting information file. DOI: 10.1107/S2056989018014020/qm21293sup8.cml

Supporting information file. DOI: 10.1107/S2056989018014020/qm21294sup9.cml

CCDC references: 1871165, 1871164, 1871163, 1871162

Additional supporting information:  crystallographic information; 3D view; checkCIF report

## Figures and Tables

**Table 1:** Distances (Å) of OMe C atoms and oxime N and O atoms from benzene ring mean plane in compounds **1**–**4**

Atom	**1**	**2**	**3**	**4** Mol A	**4** Mol B
C21	0.086 (3)	−1.140 (4)	0.195 (1)	0.121 (1)	0.059 (1)
C31	–	−0.011 (4)	–	–	–
C41	–	–	0.081 (1)	–	–
C51	–	–	–	0.033 (1)	0.061 (1)
N12	0.061 (2)	0.259 (3)	−0.177 (1)	0.264 (1)	−0.020 (1)
O13	−0.009 (2)	−0.027 (3)	0.051 (1)	0.242 (1)	0.010 (1)

**Table 2:** Hydrogen-bond geometry (Å, °) for **1** [Chem scheme1]

*D*—H⋯*A*	*D*—H	H⋯*A*	*D*⋯*A*	*D*—H⋯*A*
O13—H13⋯N12^i^	0.84	1.93	2.764 (2)	170
C3—H3⋯O13^ii^	0.95	2.50	3.442 (2)	174
C21—H21*C*⋯O13^iii^	0.98	2.57	3.506 (3)	160

Symmetry codes: (i) 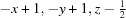; (ii) 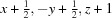; (iii) 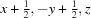.

**Table 3:** Hydrogen-bond geometry (Å, °) for **2** [Chem scheme1] *Cg*1 is the centroid of the C1–C6 ring.

*D*—H⋯*A*	*D*—H	H⋯*A*	*D*⋯*A*	*D*—H⋯*A*
O13—H13⋯N12^i^	0.97 (4)	1.87 (5)	2.805 (4)	161 (4)
C4—H4⋯O21^ii^	0.95	2.63	3.284 (4)	126
C21—H21*B*⋯O31^iii^	0.98	2.54	3.323 (5)	136
C31—H31*B*⋯O13^iv^	0.98	2.51	3.448 (5)	161
C31—H31*C*⋯*Cg*1^v^	0.98	2.73	3.599 (5)	148

Symmetry codes: (i) 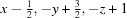; (ii) 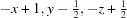; (iii) 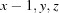; (iv) 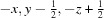; (v) 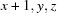.

**Table 4:** Hydrogen-bond geometry (Å, °) for **3** [Chem scheme1] *Cg*1 is the centroid of the C1–C6 ring.

*D*—H⋯*A*	*D*—H	H⋯*A*	*D*⋯*A*	*D*—H⋯*A*
O13—H13⋯N12^i^	0.893 (18)	1.995 (19)	2.8124 (13)	151.5 (15)
C41—H41*A*⋯O13^ii^	0.98	2.63	3.0680 (15)	107
C41—H41*C*⋯*Cg*1^iii^	0.98	2.60	3.4479 (13)	144

Symmetry codes: (i) 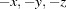; (ii) 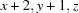; (iii) 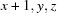.

**Table 5:** Hydrogen-bond geometry (Å, °) for **4** [Chem scheme1] *Cg*1 and *Cg*2 are the centroids of the C11–C16 and C21–C26 rings, respectively.

*D*—H⋯*A*	*D*—H	H⋯*A*	*D*⋯*A*	*D*—H⋯*A*
O113—H113⋯O121^i^	0.875 (16)	2.247 (15)	2.8944 (9)	130.7 (12)
O113—H113⋯N112^i^	0.875 (16)	1.965 (16)	2.7567 (10)	149.9 (13)
O213—H213⋯O221^ii^	0.877 (15)	2.204 (15)	2.8758 (9)	133.1 (12)
O213—H213⋯N212^ii^	0.877 (15)	2.034 (15)	2.8160 (10)	147.9 (13)
C111—H111⋯O251	0.95	2.46	3.2458 (11)	140
C121—H12*C*⋯N212^iii^	0.98	2.53	3.4400 (13)	155
C151—H15*A*⋯O113^iv^	0.98	2.50	3.3947 (11)	152
C14—H14⋯*Cg*2^iii^	0.95	2.98	3.6656 (9)	130
C151—H15*B*⋯*Cg*2	0.98	2.72	3.5973 (10)	149
C24—H24⋯*Cg*1^v^	0.95	2.67	3.4281 (10)	137
C211—H211⋯*Cg*1^vi^	0.95	2.78	3.6272 (9)	149

Symmetry codes: (i) 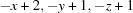; (ii) 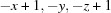; (iii) 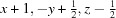; (iv) 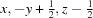; (v) 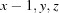; (vi) 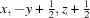.

**Table 6:** Percentages of atom–atom contacts for compounds **1**, **2**, **3** and **4** (Mol A and Mol B)

Compound	**1**	**2**	**3**	**4** Mol A	**4** Mol B
H⋯H	52.7	49.1	43.7	41.5	38.6
H⋯O/O⋯H	16.2	22.5	23.4	24.9	26.3
H⋯C/C⋯H	11.3	14.5	20.4	22.7	25.9
H⋯N/N⋯H	8.1	6.6	8.4	9.0	8.1
C⋯C	7.9	3.5	1.3	0.1	0.1
O⋯C/C⋯O	2.1	2.0	2.6	1.5	0.8
N⋯O/O⋯N	–	–	–	–	–
N⋯C/C⋯N	1.6	1.8	–	–	–
O⋯O	–	–	–	0.4	0.2

**Table 7:** Experimental details

	**1**	**2**	**3**	**4**
Crystal data				
Chemical formula	C_8_H_9_NO_2_	C_9_H_11_NO_3_	C_9_H_11_NO_3_	C_9_H_11_NO_3_
*M*_r_	151.16	181.19	181.19	181.19
Crystal system, space group	Orthorhombic, *P* *n* *a*2_1_	Orthorhombic, *P*2_1_2_1_2_1_	Triclinic, *P* 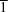	Monoclinic, *P*2_1_/*c*
Temperature (K)	100	100	100	100
*a*, *b*, *c* (Å)	11.1719 (2), 16.4260 (3), 4.0249 (1)	4.6775 (2), 13.0996 (5), 14.1984 (5)	4.9441 (2), 8.2188 (4), 12.1308 (3)	7.6480 (1), 21.3380 (4), 10.9421 (2)
α, β, γ (°)	90, 90, 90	90, 90, 90	108.849 (3), 92.288 (3), 106.273 (4)	90, 90.555 (2), 90
*V* (Å^3^)	738.61 (3)	869.98 (6)	443.17 (3)	1785.59 (5)
*Z*	4	4	2	8
Radiation type	Cu *K*α	Cu *K*α	Cu *K*α	Mo *K*α
μ (mm^−1^)	0.82	0.87	0.86	0.10
Crystal size (mm)	0.05 × 0.05 × 0.03	0.30 × 0.05 × 0.02	0.20 × 0.10 × 0.05	0.20 × 0.15 × 0.13

Data collection				
Diffractometer	Rigaku 007HF equipped with Varimax confocal mirrors and an AFC11 goniometer and HyPix 6000 detector	Rigaku 007HF equipped with Varimax confocal mirrors and an AFC11 goniometer and HyPix 6000 detector	Rigaku 007HF equipped with Varimax confocal mirrors and an AFC11 goniometer and HyPix 6000 detector	Rigaku FRE+ equipped with VHF Varimax confocal mirrors and an AFC12 goniometer and HyPix 6000 detector
Absorption correction	Multi-scan (*CrysAlis PRO*; Rigaku OD, 2017 ▸)	Multi-scan (*CrysAlis PRO*; Rigaku OD, 2017 ▸)	Multi-scan (*CrysAlis PRO*; Rigaku OD, 2017 ▸)	Multi-scan (*CrysAlis PRO*; Rigaku OD, 2017 ▸)
*T*_min_, *T*_max_	0.848, 1.000	0.507, 1.000	0.802, 1.000	0.935, 1.000
No. of measured, independent and observed [*I* > 2σ(*I*)] reflections	12857, 1345, 1325	7835, 1596, 1371	7618, 1594, 1462	38753, 4082, 3761
*R*_int_	0.038	0.095	0.033	0.020
(sin θ/λ)_max_ (Å^−1^)	0.602	0.602	0.602	0.649

Refinement				
*R*[*F* ^2^ > 2σ(*F* ^2^)], *wR*(*F* ^2^), *S*	0.033, 0.088, 1.08	0.058, 0.151, 1.04	0.035, 0.101, 0.88	0.031, 0.086, 1.06
No. of reflections	1345	1596	1594	4082
No. of parameters	102	124	124	247
No. of restraints	1	0	0	0
H-atom treatment	H-atom parameters constrained	H atoms treated by a mixture of independent and constrained refinement	H atoms treated by a mixture of independent and constrained refinement	H atoms treated by a mixture of independent and constrained refinement
Δρ_max_, Δρ_min_ (e Å^−3^)	0.17, −0.16	0.35, −0.20	0.20, −0.19	0.32, −0.19
Absolute structure	Refined as a perfect inversion twin.	Flack *x* determined using 474 quotients [(*I* ^+^)−(*I* ^−^)]/[(*I* ^+^)+(*I* ^−^)] (Parsons *et al.*, 2013 ▸)	–	–
Absolute structure parameter	0.5	0.2 (3)	–	–

Computer programs: *CrysAlis PRO* (Rigaku OD, 2017 ▸), *OSCAIL* (McArdle *et al.*, 2004 ▸), *SHELXT* (Sheldrick, 2015*a* ▸), *ShelXle* (Hübschle *et al.*, 2011 ▸), *SHELXL* (Sheldrick, 2015*b* ▸), *Mercury* (Macrae *et al.*, 2006 ▸) and *PLATON* (Spek, 2009 ▸).

## supplementary crystallographic information

### 2-Methoxy-benzaldehyde oxime (1) Crystal data

**Table:**

C_8_H_9_NO_2_	*D*_x_ = 1.359 Mg m^−^^3^
*M**_r_* = 151.16	Cu *K*α radiation, λ = 1.54178 Å
Orthorhombic, *P**n**a*2_1_	Cell parameters from 7376 reflections
*a* = 11.1719 (2) Å	θ = 2.7–70.0°
*b* = 16.4260 (3) Å	µ = 0.82 mm^−^^1^
*c* = 4.0249 (1) Å	*T* = 100 K
*V* = 738.61 (3) Å^3^	Block, colourless
*Z* = 4	0.05 × 0.05 × 0.03 mm
*F*(000) = 320	

### 2-Methoxy-benzaldehyde oxime (1) Data collection

**Table:**

Rigaku 007HF equipped with Varimax confocal mirrors and an AFC11 goniometer and HyPix 6000 detector diffractometer	1345 independent reflections
Radiation source: Rotating anode, Rigaku 007 HF	1325 reflections with *I* > 2σ(*I*)
Detector resolution: 10 pixels mm^-1^	*R*_int_ = 0.038
profile data from ω–scans	θ_max_ = 68.2°, θ_min_ = 4.8°
Absorption correction: multi-scan (CrysAlis PRO; Rigaku OD, 2017)	*h* = −13→13
*T*_min_ = 0.848, *T*_max_ = 1.000	*k* = −19→19
12857 measured reflections	*l* = −4→4

### 2-Methoxy-benzaldehyde oxime (1) Refinement

**Table:**

Refinement on *F*^2^	Hydrogen site location: inferred from neighbouring sites
Least-squares matrix: full	H-atom parameters constrained
*R*[*F*^2^ > 2σ(*F*^2^)] = 0.033	*w* = 1/[σ^2^(*F*_o_^2^) + (0.0559*P*)^2^ + 0.1277*P*] where *P* = (*F*_o_^2^ + 2*F*_c_^2^)/3
*wR*(*F*^2^) = 0.088	(Δ/σ)_max_ < 0.001
*S* = 1.08	Δρ_max_ = 0.17 e Å^−^^3^
1345 reflections	Δρ_min_ = −0.16 e Å^−^^3^
102 parameters	Absolute structure: Refined as a perfect inversion twin.
1 restraint	Absolute structure parameter: 0.5

### 2-Methoxy-benzaldehyde oxime (1) Special details

**Table:**

Geometry. All esds (except the esd in the dihedral angle between two l.s. planes) are estimated using the full covariance matrix. The cell esds are taken into account individually in the estimation of esds in distances, angles and torsion angles; correlations between esds in cell parameters are only used when they are defined by crystal symmetry. An approximate (isotropic) treatment of cell esds is used for estimating esds involving l.s. planes.
Refinement. Refined as a 2-component perfect inversion twin.

### 2-Methoxy-benzaldehyde oxime (1) Fractional atomic coordinates and isotropic or equivalent isotropic displacement parameters (Å^2^)

**Table:**

	*x*	*y*	*z*	*U*_iso_*/*U*_eq_	
O13	0.45979 (12)	0.42019 (8)	−0.4095 (4)	0.0311 (4)	
H13	0.443792	0.464123	−0.506595	0.047*	
O21	0.69333 (12)	0.21322 (8)	0.0350 (4)	0.0303 (4)	
N12	0.56325 (15)	0.43011 (9)	−0.2166 (5)	0.0265 (4)	
C1	0.72029 (18)	0.35490 (12)	0.0488 (5)	0.0260 (5)	
C2	0.76289 (18)	0.27720 (11)	0.1386 (6)	0.0266 (5)	
C3	0.86797 (18)	0.26830 (12)	0.3172 (6)	0.0296 (5)	
H3	0.895987	0.215571	0.375132	0.036*	
C4	0.93229 (19)	0.33707 (13)	0.4113 (6)	0.0316 (5)	
H4	1.004194	0.331184	0.535045	0.038*	
C5	0.89200 (18)	0.41434 (12)	0.3255 (6)	0.0310 (5)	
H5	0.936169	0.461141	0.390355	0.037*	
C6	0.78780 (18)	0.42272 (12)	0.1462 (6)	0.0296 (5)	
H6	0.761062	0.475674	0.087081	0.035*	
C11	0.61087 (18)	0.36135 (11)	−0.1461 (6)	0.0275 (5)	
H11	0.573609	0.312951	−0.223553	0.033*	
C21	0.7290 (2)	0.13337 (11)	0.1388 (7)	0.0319 (5)	
H21A	0.668356	0.093725	0.069500	0.048*	
H21B	0.736939	0.132217	0.381231	0.048*	
H21C	0.805963	0.119570	0.036670	0.048*	

### 2-Methoxy-benzaldehyde oxime (1) Atomic displacement parameters (Å^2^)

**Table:**

	*U*^11^	*U*^22^	*U*^33^	*U*^12^	*U*^13^	*U*^23^
O13	0.0346 (7)	0.0199 (6)	0.0389 (9)	−0.0001 (6)	−0.0067 (6)	0.0040 (6)
O21	0.0346 (7)	0.0172 (7)	0.0391 (9)	0.0000 (5)	−0.0025 (7)	0.0018 (6)
N12	0.0300 (8)	0.0215 (8)	0.0279 (9)	0.0012 (6)	0.0017 (7)	−0.0006 (7)
C1	0.0310 (10)	0.0206 (9)	0.0264 (11)	0.0008 (7)	0.0041 (9)	−0.0005 (8)
C2	0.0323 (9)	0.0194 (9)	0.0281 (11)	−0.0011 (7)	0.0048 (9)	−0.0007 (8)
C3	0.0348 (10)	0.0230 (9)	0.0310 (11)	0.0017 (8)	0.0036 (9)	0.0015 (9)
C4	0.0316 (10)	0.0307 (11)	0.0324 (12)	0.0001 (8)	0.0009 (9)	0.0006 (9)
C5	0.0350 (11)	0.0243 (10)	0.0337 (12)	−0.0043 (7)	0.0015 (9)	−0.0025 (10)
C6	0.0362 (11)	0.0203 (9)	0.0322 (11)	0.0003 (8)	0.0023 (9)	−0.0007 (9)
C11	0.0352 (11)	0.0181 (8)	0.0292 (11)	−0.0008 (7)	0.0030 (9)	−0.0011 (8)
C21	0.0390 (11)	0.0158 (9)	0.0408 (12)	0.0028 (7)	−0.0004 (10)	0.0028 (9)

### 2-Methoxy-benzaldehyde oxime (1) Geometric parameters (Å, º)

**Table:**

O13—N12	1.402 (2)	C3—H3	0.9500
O13—H13	0.8400	C4—C5	1.390 (3)
O21—C2	1.372 (2)	C4—H4	0.9500
O21—C21	1.433 (2)	C5—C6	1.377 (3)
N12—C11	1.280 (3)	C5—H5	0.9500
C1—C6	1.401 (3)	C6—H6	0.9500
C1—C2	1.409 (3)	C11—H11	0.9500
C1—C11	1.456 (3)	C21—H21A	0.9800
C2—C3	1.384 (3)	C21—H21B	0.9800
C3—C4	1.391 (3)	C21—H21C	0.9800
			
N12—O13—H13	109.5	C6—C5—C4	119.69 (18)
C2—O21—C21	117.07 (16)	C6—C5—H5	120.2
C11—N12—O13	111.27 (15)	C4—C5—H5	120.2
C6—C1—C2	117.80 (19)	C5—C6—C1	121.50 (18)
C6—C1—C11	123.00 (17)	C5—C6—H6	119.3
C2—C1—C11	119.18 (17)	C1—C6—H6	119.3
O21—C2—C3	123.87 (17)	N12—C11—C1	122.16 (18)
O21—C2—C1	115.11 (18)	N12—C11—H11	118.9
C3—C2—C1	121.01 (17)	C1—C11—H11	118.9
C2—C3—C4	119.58 (18)	O21—C21—H21A	109.5
C2—C3—H3	120.2	O21—C21—H21B	109.5
C4—C3—H3	120.2	H21A—C21—H21B	109.5
C5—C4—C3	120.4 (2)	O21—C21—H21C	109.5
C5—C4—H4	119.8	H21A—C21—H21C	109.5
C3—C4—H4	119.8	H21B—C21—H21C	109.5
			
C21—O21—C2—C3	4.2 (3)	C2—C3—C4—C5	−0.4 (3)
C21—O21—C2—C1	−176.10 (18)	C3—C4—C5—C6	0.0 (3)
C6—C1—C2—O21	−179.70 (18)	C4—C5—C6—C1	0.4 (3)
C11—C1—C2—O21	−1.2 (3)	C2—C1—C6—C5	−0.4 (3)
C6—C1—C2—C3	0.0 (3)	C11—C1—C6—C5	−178.8 (2)
C11—C1—C2—C3	178.5 (2)	O13—N12—C11—C1	179.25 (17)
O21—C2—C3—C4	−179.9 (2)	C6—C1—C11—N12	−6.4 (3)
C1—C2—C3—C4	0.4 (3)	C2—C1—C11—N12	175.2 (2)

### 2-Methoxy-benzaldehyde oxime (1) Hydrogen-bond geometry (Å, º)

**Table:**

*D*—H···*A*	*D*—H	H···*A*	*D*···*A*	*D*—H···*A*
O13—H13···N12^i^	0.84	1.93	2.764 (2)	170
C3—H3···O13^ii^	0.95	2.50	3.442 (2)	174
C21—H21*C*···O13^iii^	0.98	2.57	3.506 (3)	160

Symmetry codes: (i) −*x*+1, −*y*+1, *z*−1/2; (ii) *x*+1/2, −*y*+1/2, *z*+1; (iii) *x*+1/2, −*y*+1/2, *z*.

### 2,3-Dimethoxy-benzaldehyde oxime (2) Crystal data

**Table:**

C_9_H_11_NO_3_	*D*_x_ = 1.383 Mg m^−^^3^
*M**_r_* = 181.19	Cu *K*α radiation, λ = 1.54178 Å
Orthorhombic, *P*2_1_2_1_2_1_	Cell parameters from 2093 reflections
*a* = 4.6775 (2) Å	θ = 4.6–67.5°
*b* = 13.0996 (5) Å	µ = 0.87 mm^−^^1^
*c* = 14.1984 (5) Å	*T* = 100 K
*V* = 869.98 (6) Å^3^	Needle, colourless
*Z* = 4	0.30 × 0.05 × 0.02 mm
*F*(000) = 384	

### 2,3-Dimethoxy-benzaldehyde oxime (2) Data collection

**Table:**

Rigaku 007HF equipped with Varimax confocal mirrors and an AFC11 goniometer and HyPix 6000 detector diffractometer	1596 independent reflections
Radiation source: Rotating anode, Rigaku 007 HF	1371 reflections with *I* > 2σ(*I*)
Detector resolution: 10 pixels mm^-1^	*R*_int_ = 0.095
profile data from ω–scans	θ_max_ = 68.2°, θ_min_ = 4.6°
Absorption correction: multi-scan (CrysAlis PRO; Rigaku OD, 2017)	*h* = −5→5
*T*_min_ = 0.507, *T*_max_ = 1.000	*k* = −15→15
7835 measured reflections	*l* = −17→16

### 2,3-Dimethoxy-benzaldehyde oxime (2) Refinement

**Table:**

Refinement on *F*^2^	Hydrogen site location: mixed
Least-squares matrix: full	H atoms treated by a mixture of independent and constrained refinement
*R*[*F*^2^ > 2σ(*F*^2^)] = 0.058	*w* = 1/[σ^2^(*F*_o_^2^) + (0.1064*P*)^2^] where *P* = (*F*_o_^2^ + 2*F*_c_^2^)/3
*wR*(*F*^2^) = 0.151	(Δ/σ)_max_ < 0.001
*S* = 1.03	Δρ_max_ = 0.35 e Å^−^^3^
1596 reflections	Δρ_min_ = −0.20 e Å^−^^3^
124 parameters	Absolute structure: Flack *x* determined using 474 quotients [(*I*^+^)-(*I*^-^)]/[(*I*^+^)+(*I*^-^)] (Parsons *et al.*, 2013)
0 restraints	Absolute structure parameter: 0.2 (3)

### 2,3-Dimethoxy-benzaldehyde oxime (2) Special details

**Table:**

Geometry. All esds (except the esd in the dihedral angle between two l.s. planes) are estimated using the full covariance matrix. The cell esds are taken into account individually in the estimation of esds in distances, angles and torsion angles; correlations between esds in cell parameters are only used when they are defined by crystal symmetry. An approximate (isotropic) treatment of cell esds is used for estimating esds involving l.s. planes.

### 2,3-Dimethoxy-benzaldehyde oxime (2) Fractional atomic coordinates and isotropic or equivalent isotropic displacement parameters (Å^2^)

**Table:**

	*x*	*y*	*z*	*U*_iso_*/*U*_eq_	
O13	−0.3161 (6)	0.81289 (19)	0.42174 (19)	0.0376 (7)	
H13	−0.382 (10)	0.799 (3)	0.485 (3)	0.045 (12)*	
O21	0.3629 (5)	0.73710 (18)	0.18056 (18)	0.0331 (6)	
O31	0.6683 (6)	0.57595 (18)	0.1198 (2)	0.0370 (7)	
N12	−0.1250 (7)	0.7305 (2)	0.4137 (2)	0.0328 (7)	
C1	0.1671 (8)	0.6379 (3)	0.3072 (3)	0.0311 (8)	
C2	0.3440 (8)	0.6450 (2)	0.2283 (3)	0.0309 (8)	
C3	0.5135 (8)	0.5622 (2)	0.1997 (3)	0.0315 (8)	
C4	0.5069 (9)	0.4725 (3)	0.2525 (2)	0.0331 (9)	
H4	0.620619	0.415756	0.234431	0.040*	
C5	0.3334 (9)	0.4664 (3)	0.3318 (3)	0.0344 (8)	
H5	0.332576	0.405472	0.368039	0.041*	
C6	0.1631 (9)	0.5468 (3)	0.3588 (3)	0.0338 (9)	
H6	0.042859	0.540487	0.412457	0.041*	
C11	−0.0229 (8)	0.7232 (2)	0.3309 (3)	0.0332 (8)	
H11	−0.069638	0.772807	0.284541	0.040*	
C21	0.2048 (9)	0.7399 (3)	0.0942 (3)	0.0374 (9)	
H21A	0.230310	0.806598	0.064099	0.056*	
H21B	0.001542	0.728827	0.107410	0.056*	
H21C	0.274481	0.686165	0.052004	0.056*	
C31	0.8511 (10)	0.4933 (3)	0.0919 (3)	0.0390 (10)	
H31A	0.952242	0.511808	0.033893	0.059*	
H31B	0.735578	0.432080	0.080781	0.059*	
H31C	0.990297	0.479650	0.141905	0.059*	

### 2,3-Dimethoxy-benzaldehyde oxime (2) Atomic displacement parameters (Å^2^)

**Table:**

	*U*^11^	*U*^22^	*U*^33^	*U*^12^	*U*^13^	*U*^23^
O13	0.0411 (16)	0.0292 (13)	0.0423 (16)	0.0088 (12)	0.0053 (13)	0.0027 (11)
O21	0.0339 (14)	0.0250 (12)	0.0405 (14)	−0.0027 (11)	0.0009 (12)	0.0020 (10)
O31	0.0340 (14)	0.0291 (12)	0.0478 (15)	0.0042 (11)	0.0072 (13)	−0.0015 (11)
N12	0.0294 (16)	0.0244 (14)	0.0446 (18)	0.0025 (13)	−0.0001 (15)	−0.0001 (12)
C1	0.0277 (18)	0.0277 (17)	0.038 (2)	−0.0008 (15)	−0.0021 (17)	−0.0004 (14)
C2	0.0297 (17)	0.0244 (17)	0.0387 (19)	−0.0018 (15)	−0.0012 (17)	0.0017 (14)
C3	0.0285 (18)	0.0262 (16)	0.040 (2)	0.0016 (15)	0.0002 (16)	−0.0029 (15)
C4	0.0315 (18)	0.0236 (17)	0.044 (2)	0.0017 (16)	−0.0025 (17)	−0.0036 (14)
C5	0.036 (2)	0.0286 (17)	0.039 (2)	0.0000 (16)	−0.0054 (18)	0.0019 (15)
C6	0.034 (2)	0.0298 (18)	0.038 (2)	0.0001 (17)	−0.0004 (17)	0.0019 (15)
C11	0.0353 (19)	0.0238 (16)	0.040 (2)	0.0010 (17)	0.0001 (18)	0.0007 (15)
C21	0.040 (2)	0.0324 (18)	0.040 (2)	−0.0016 (17)	0.0010 (17)	0.0037 (15)
C31	0.035 (2)	0.0296 (18)	0.052 (2)	0.0034 (16)	0.009 (2)	−0.0068 (16)

### 2,3-Dimethoxy-benzaldehyde oxime (2) Geometric parameters (Å, º)

**Table:**

O13—N12	1.405 (4)	C4—C5	1.389 (5)
O13—H13	0.97 (4)	C4—H4	0.9500
O21—C2	1.386 (4)	C5—C6	1.375 (5)
O21—C21	1.432 (4)	C5—H5	0.9500
O31—C3	1.357 (4)	C6—H6	0.9500
O31—C31	1.435 (4)	C11—H11	0.9500
N12—C11	1.273 (5)	C21—H21A	0.9800
C1—C2	1.396 (5)	C21—H21B	0.9800
C1—C6	1.401 (5)	C21—H21C	0.9800
C1—C11	1.467 (5)	C31—H31A	0.9800
C2—C3	1.404 (5)	C31—H31B	0.9800
C3—C4	1.394 (5)	C31—H31C	0.9800
			
N12—O13—H13	97 (3)	C5—C6—C1	119.9 (4)
C2—O21—C21	114.1 (3)	C5—C6—H6	120.1
C3—O31—C31	116.6 (3)	C1—C6—H6	120.1
C11—N12—O13	111.8 (3)	N12—C11—C1	119.7 (3)
C2—C1—C6	119.0 (3)	N12—C11—H11	120.1
C2—C1—C11	119.5 (3)	C1—C11—H11	120.1
C6—C1—C11	121.4 (3)	O21—C21—H21A	109.5
O21—C2—C1	119.2 (3)	O21—C21—H21B	109.5
O21—C2—C3	119.7 (3)	H21A—C21—H21B	109.5
C1—C2—C3	121.0 (3)	O21—C21—H21C	109.5
O31—C3—C4	125.0 (3)	H21A—C21—H21C	109.5
O31—C3—C2	116.1 (3)	H21B—C21—H21C	109.5
C4—C3—C2	118.9 (3)	O31—C31—H31A	109.5
C5—C4—C3	119.8 (3)	O31—C31—H31B	109.5
C5—C4—H4	120.1	H31A—C31—H31B	109.5
C3—C4—H4	120.1	O31—C31—H31C	109.5
C6—C5—C4	121.4 (3)	H31A—C31—H31C	109.5
C6—C5—H5	119.3	H31B—C31—H31C	109.5
C4—C5—H5	119.3		
			
C21—O21—C2—C1	−103.3 (4)	C1—C2—C3—C4	−1.2 (5)
C21—O21—C2—C3	80.0 (4)	O31—C3—C4—C5	−178.1 (4)
C6—C1—C2—O21	−175.9 (3)	C2—C3—C4—C5	0.2 (5)
C11—C1—C2—O21	7.9 (5)	C3—C4—C5—C6	1.1 (6)
C6—C1—C2—C3	0.8 (5)	C4—C5—C6—C1	−1.4 (6)
C11—C1—C2—C3	−175.4 (4)	C2—C1—C6—C5	0.5 (6)
C31—O31—C3—C4	−4.0 (5)	C11—C1—C6—C5	176.6 (3)
C31—O31—C3—C2	177.6 (4)	O13—N12—C11—C1	−176.2 (3)
O21—C2—C3—O31	−6.0 (5)	C2—C1—C11—N12	−161.0 (3)
C1—C2—C3—O31	177.3 (3)	C6—C1—C11—N12	22.8 (6)
O21—C2—C3—C4	175.5 (3)		

### 2,3-Dimethoxy-benzaldehyde oxime (2) Hydrogen-bond geometry (Å, º)

Cg1 is the centroid of the C1–C6 ring.

**Table:**

*D*—H···*A*	*D*—H	H···*A*	*D*···*A*	*D*—H···*A*
O13—H13···N12^i^	0.97 (4)	1.87 (5)	2.805 (4)	161 (4)
C4—H4···O21^ii^	0.95	2.63	3.284 (4)	126
C21—H21*B*···O31^iii^	0.98	2.54	3.323 (5)	136
C31—H31*B*···O13^iv^	0.98	2.51	3.448 (5)	161
C31—H31*C*···*Cg*1^v^	0.98	2.73	3.599 (5)	148

Symmetry codes: (i) *x*−1/2, −*y*+3/2, −*z*+1; (ii) −*x*+1, *y*−1/2, −*z*+1/2; (iii) *x*−1, *y*, *z*; (iv) −*x*, *y*−1/2, −*z*+1/2; (v) *x*+1, *y*, *z*.

### 2,4-Dimethoxybenzaldehyde oxime (3) Crystal data

**Table:**

C_9_H_11_NO_3_	*Z* = 2
*M**_r_* = 181.19	*F*(000) = 192
Triclinic, *P*1	*D*_x_ = 1.358 Mg m^−^^3^
*a* = 4.9441 (2) Å	Cu *K*α radiation, λ = 1.54178 Å
*b* = 8.2188 (4) Å	Cell parameters from 3758 reflections
*c* = 12.1308 (3) Å	θ = 3.9–69.9°
α = 108.849 (3)°	µ = 0.86 mm^−^^1^
β = 92.288 (3)°	*T* = 100 K
γ = 106.273 (4)°	Block, colourless
*V* = 443.17 (3) Å^3^	0.20 × 0.10 × 0.05 mm

### 2,4-Dimethoxybenzaldehyde oxime (3) Data collection

**Table:**

Rigaku 007HF equipped with Varimax confocal mirrors and an AFC11 goniometer and HyPix 6000 detector diffractometer	1594 independent reflections
Radiation source: Rotating anode, Rigaku 007 HF	1462 reflections with *I* > 2σ(*I*)
Detector resolution: 10 pixels mm^-1^	*R*_int_ = 0.033
profile data from ω–scans	θ_max_ = 68.2°, θ_min_ = 3.9°
Absorption correction: multi-scan (CrysAlis PRO; Rigaku OD, 2017)	*h* = −5→5
*T*_min_ = 0.802, *T*_max_ = 1.000	*k* = −9→9
7618 measured reflections	*l* = −14→13

### 2,4-Dimethoxybenzaldehyde oxime (3) Refinement

**Table:**

Refinement on *F*^2^	0 restraints
Least-squares matrix: full	Hydrogen site location: mixed
*R*[*F*^2^ > 2σ(*F*^2^)] = 0.035	H atoms treated by a mixture of independent and constrained refinement
*wR*(*F*^2^) = 0.101	*w* = 1/[σ^2^(*F*_o_^2^) + (0.0775*P*)^2^ + 0.1273*P*] where *P* = (*F*_o_^2^ + 2*F*_c_^2^)/3
*S* = 0.88	(Δ/σ)_max_ < 0.001
1594 reflections	Δρ_max_ = 0.20 e Å^−^^3^
124 parameters	Δρ_min_ = −0.19 e Å^−^^3^

### 2,4-Dimethoxybenzaldehyde oxime (3) Special details

**Table:**

Geometry. All esds (except the esd in the dihedral angle between two l.s. planes) are estimated using the full covariance matrix. The cell esds are taken into account individually in the estimation of esds in distances, angles and torsion angles; correlations between esds in cell parameters are only used when they are defined by crystal symmetry. An approximate (isotropic) treatment of cell esds is used for estimating esds involving l.s. planes.

### 2,4-Dimethoxybenzaldehyde oxime (3) Fractional atomic coordinates and isotropic or equivalent isotropic displacement parameters (Å^2^)

**Table:**

	*x*	*y*	*z*	*U*_iso_*/*U*_eq_	
O13	−0.22417 (16)	0.06653 (12)	0.08642 (7)	0.0311 (2)	
O21	0.29524 (16)	0.56560 (10)	0.41358 (7)	0.0261 (2)	
O41	1.14895 (16)	0.88605 (11)	0.31075 (7)	0.0273 (2)	
N12	0.05891 (19)	0.17759 (13)	0.09919 (8)	0.0255 (3)	
C1	0.3951 (2)	0.45758 (15)	0.22073 (10)	0.0231 (3)	
C2	0.4791 (2)	0.58860 (15)	0.33490 (9)	0.0228 (3)	
C3	0.7331 (2)	0.72870 (15)	0.36234 (9)	0.0237 (3)	
H3	0.789071	0.815115	0.439846	0.028*	
C4	0.9063 (2)	0.74213 (15)	0.27541 (10)	0.0238 (3)	
C5	0.8276 (2)	0.61490 (15)	0.16177 (10)	0.0247 (3)	
H5	0.945380	0.624026	0.102665	0.030*	
C6	0.5741 (2)	0.47475 (15)	0.13657 (10)	0.0245 (3)	
H6	0.520758	0.387495	0.059294	0.029*	
C11	0.1194 (2)	0.31641 (15)	0.19252 (10)	0.0242 (3)	
H11	−0.017316	0.328624	0.244952	0.029*	
C21	0.3513 (2)	0.70704 (15)	0.52560 (9)	0.0281 (3)	
H21A	0.196509	0.679099	0.571095	0.042*	
H21B	0.364539	0.821605	0.514202	0.042*	
H21C	0.531398	0.717369	0.568289	0.042*	
C41	1.3260 (2)	0.90933 (16)	0.22272 (10)	0.0278 (3)	
H41A	1.490351	1.018155	0.257704	0.042*	
H41B	1.216391	0.922054	0.158580	0.042*	
H41C	1.392155	0.803696	0.191690	0.042*	
H13	−0.235 (4)	−0.031 (2)	0.0244 (16)	0.049 (5)*	

### 2,4-Dimethoxybenzaldehyde oxime (3) Atomic displacement parameters (Å^2^)

**Table:**

	*U*^11^	*U*^22^	*U*^33^	*U*^12^	*U*^13^	*U*^23^
O13	0.0234 (4)	0.0320 (5)	0.0287 (5)	−0.0008 (3)	0.0076 (3)	0.0062 (4)
O21	0.0251 (4)	0.0298 (4)	0.0202 (4)	0.0037 (3)	0.0072 (3)	0.0081 (3)
O41	0.0227 (4)	0.0309 (5)	0.0241 (4)	0.0022 (3)	0.0068 (3)	0.0089 (3)
N12	0.0212 (5)	0.0278 (5)	0.0252 (5)	0.0032 (4)	0.0044 (4)	0.0100 (4)
C1	0.0229 (6)	0.0248 (6)	0.0230 (6)	0.0079 (4)	0.0033 (4)	0.0099 (4)
C2	0.0222 (5)	0.0285 (6)	0.0216 (6)	0.0092 (4)	0.0060 (4)	0.0123 (5)
C3	0.0235 (6)	0.0279 (6)	0.0196 (5)	0.0072 (5)	0.0034 (4)	0.0086 (4)
C4	0.0198 (6)	0.0269 (6)	0.0257 (6)	0.0063 (4)	0.0029 (4)	0.0114 (5)
C5	0.0236 (6)	0.0306 (6)	0.0229 (6)	0.0100 (5)	0.0077 (4)	0.0113 (5)
C6	0.0252 (6)	0.0271 (6)	0.0208 (5)	0.0083 (5)	0.0036 (4)	0.0077 (4)
C11	0.0242 (6)	0.0288 (6)	0.0221 (6)	0.0080 (5)	0.0058 (4)	0.0118 (4)
C21	0.0295 (6)	0.0324 (6)	0.0195 (6)	0.0065 (5)	0.0077 (4)	0.0075 (5)
C41	0.0250 (6)	0.0333 (6)	0.0258 (6)	0.0059 (5)	0.0085 (4)	0.0131 (5)

### 2,4-Dimethoxybenzaldehyde oxime (3) Geometric parameters (Å, º)

**Table:**

O13—N12	1.4112 (12)	C3—H3	0.9500
O13—H13	0.893 (18)	C4—C5	1.3939 (16)
O21—C2	1.3648 (13)	C5—C6	1.3869 (16)
O21—C21	1.4294 (13)	C5—H5	0.9500
O41—C4	1.3632 (13)	C6—H6	0.9500
O41—C41	1.4339 (13)	C11—H11	0.9500
N12—C11	1.2728 (15)	C21—H21A	0.9800
C1—C6	1.3931 (16)	C21—H21B	0.9800
C1—C2	1.4107 (15)	C21—H21C	0.9800
C1—C11	1.4634 (15)	C41—H41A	0.9800
C2—C3	1.3864 (16)	C41—H41B	0.9800
C3—C4	1.3971 (16)	C41—H41C	0.9800
			
N12—O13—H13	103.5 (11)	C1—C6—C5	122.23 (10)
C2—O21—C21	117.44 (8)	C1—C6—H6	118.9
C4—O41—C41	116.82 (9)	C5—C6—H6	118.9
C11—N12—O13	111.40 (9)	N12—C11—C1	121.37 (10)
C6—C1—C2	117.84 (10)	N12—C11—H11	119.3
C6—C1—C11	122.31 (10)	C1—C11—H11	119.3
C2—C1—C11	119.74 (10)	O21—C21—H21A	109.5
O21—C2—C3	123.79 (10)	O21—C21—H21B	109.5
O21—C2—C1	115.34 (10)	H21A—C21—H21B	109.5
C3—C2—C1	120.88 (10)	O21—C21—H21C	109.5
C2—C3—C4	119.64 (10)	H21A—C21—H21C	109.5
C2—C3—H3	120.2	H21B—C21—H21C	109.5
C4—C3—H3	120.2	O41—C41—H41A	109.5
O41—C4—C3	115.11 (10)	O41—C41—H41B	109.5
O41—C4—C5	124.27 (10)	H41A—C41—H41B	109.5
C3—C4—C5	120.62 (10)	O41—C41—H41C	109.5
C6—C5—C4	118.78 (10)	H41A—C41—H41C	109.5
C6—C5—H5	120.6	H41B—C41—H41C	109.5
C4—C5—H5	120.6		
			
C21—O21—C2—C3	8.36 (15)	C2—C3—C4—O41	179.03 (9)
C21—O21—C2—C1	−171.96 (9)	C2—C3—C4—C5	−0.70 (17)
C6—C1—C2—O21	179.72 (9)	O41—C4—C5—C6	−179.69 (9)
C11—C1—C2—O21	3.38 (15)	C3—C4—C5—C6	0.01 (17)
C6—C1—C2—C3	−0.59 (16)	C2—C1—C6—C5	−0.11 (17)
C11—C1—C2—C3	−176.93 (9)	C11—C1—C6—C5	176.12 (9)
O21—C2—C3—C4	−179.34 (9)	C4—C5—C6—C1	0.40 (17)
C1—C2—C3—C4	1.00 (16)	O13—N12—C11—C1	−175.95 (9)
C41—O41—C4—C3	−177.21 (9)	C6—C1—C11—N12	17.32 (17)
C41—O41—C4—C5	2.51 (16)	C2—C1—C11—N12	−166.51 (10)

### 2,4-Dimethoxybenzaldehyde oxime (3) Hydrogen-bond geometry (Å, º)

Cg1 is the centroid of the C1–C6 ring.

**Table:**

*D*—H···*A*	*D*—H	H···*A*	*D*···*A*	*D*—H···*A*
O13—H13···N12^i^	0.893 (18)	1.995 (19)	2.8124 (13)	151.5 (15)
C41—H41*A*···O13^ii^	0.98	2.63	3.0680 (15)	107
C41—H41*C*···*Cg*1^iii^	0.98	2.60	3.4479 (13)	144

Symmetry codes: (i) −*x*, −*y*, −*z*; (ii) *x*+2, *y*+1, *z*; (iii) *x*+1, *y*, *z*.

### 2,5-Dimethoxybenzaldehyde oxime (4) Crystal data

**Table:**

C_9_H_11_NO_3_	*F*(000) = 768
*M**_r_* = 181.19	*D*_x_ = 1.348 Mg m^−^^3^
Monoclinic, *P*2_1_/*c*	Mo *K*α radiation, λ = 0.71075 Å
*a* = 7.6480 (1) Å	Cell parameters from 21005 reflections
*b* = 21.3380 (4) Å	θ = 2.1–32.1°
*c* = 10.9421 (2) Å	µ = 0.10 mm^−^^1^
β = 90.555 (2)°	*T* = 100 K
*V* = 1785.59 (5) Å^3^	Block, colourless
*Z* = 8	0.20 × 0.15 × 0.13 mm

### 2,5-Dimethoxybenzaldehyde oxime (4) Data collection

**Table:**

Rigaku FRE+ equipped with VHF Varimax confocal mirrors and an AFC12 goniometer and HyPix 6000 detector diffractometer	4082 independent reflections
Radiation source: Rotating Anode, Rigaku FRE+	3761 reflections with *I* > 2σ(*I*)
Confocal mirrors, VHF Varimax monochromator	*R*_int_ = 0.020
Detector resolution: 10 pixels mm^-1^	θ_max_ = 27.5°, θ_min_ = 1.9°
profile data from ω–scans	*h* = −9→9
Absorption correction: multi-scan (CrysAlis PRO; Rigaku OD, 2017)	*k* = −27→27
*T*_min_ = 0.935, *T*_max_ = 1.000	*l* = −14→14
38753 measured reflections	

### 2,5-Dimethoxybenzaldehyde oxime (4) Refinement

**Table:**

Refinement on *F*^2^	0 restraints
Least-squares matrix: full	Hydrogen site location: mixed
*R*[*F*^2^ > 2σ(*F*^2^)] = 0.031	H atoms treated by a mixture of independent and constrained refinement
*wR*(*F*^2^) = 0.086	*w* = 1/[σ^2^(*F*_o_^2^) + (0.0477*P*)^2^ + 0.5056*P*] where *P* = (*F*_o_^2^ + 2*F*_c_^2^)/3
*S* = 1.06	(Δ/σ)_max_ = 0.001
4082 reflections	Δρ_max_ = 0.32 e Å^−^^3^
247 parameters	Δρ_min_ = −0.19 e Å^−^^3^

### 2,5-Dimethoxybenzaldehyde oxime (4) Special details

**Table:**

Geometry. All esds (except the esd in the dihedral angle between two l.s. planes) are estimated using the full covariance matrix. The cell esds are taken into account individually in the estimation of esds in distances, angles and torsion angles; correlations between esds in cell parameters are only used when they are defined by crystal symmetry. An approximate (isotropic) treatment of cell esds is used for estimating esds involving l.s. planes.

### 2,5-Dimethoxybenzaldehyde oxime (4) Fractional atomic coordinates and isotropic or equivalent isotropic displacement parameters (Å^2^)

**Table:**

	*x*	*y*	*z*	*U*_iso_*/*U*_eq_	
O121	1.05737 (9)	0.46081 (3)	0.23188 (6)	0.01640 (15)	
O221	0.33772 (9)	0.09728 (3)	0.32374 (6)	0.01705 (15)	
O151	0.88735 (9)	0.21358 (3)	0.13343 (6)	0.01629 (14)	
O213	0.63115 (9)	0.03239 (3)	0.60782 (6)	0.01852 (15)	
H213	0.6028 (19)	−0.0064 (7)	0.5898 (13)	0.036 (4)*	
O251	0.47197 (9)	0.34248 (3)	0.46029 (6)	0.01810 (15)	
O113	0.84907 (9)	0.45697 (3)	0.56752 (6)	0.01890 (15)	
H113	0.905 (2)	0.4914 (7)	0.5868 (13)	0.036 (4)*	
N112	0.92134 (10)	0.44408 (4)	0.45300 (7)	0.01543 (16)	
N212	0.53640 (10)	0.06589 (4)	0.51894 (7)	0.01507 (16)	
C11	0.92057 (11)	0.36690 (4)	0.29191 (8)	0.01265 (17)	
C12	1.01564 (11)	0.40037 (4)	0.20387 (8)	0.01345 (17)	
C13	1.06269 (12)	0.37068 (4)	0.09534 (8)	0.01520 (18)	
H13	1.125790	0.393201	0.035247	0.018*	
C14	1.01792 (11)	0.30839 (4)	0.07452 (8)	0.01529 (18)	
H14	1.051935	0.288508	0.000789	0.018*	
C15	0.92359 (11)	0.27498 (4)	0.16103 (8)	0.01354 (17)	
C16	0.87441 (11)	0.30442 (4)	0.26854 (8)	0.01321 (17)	
H16	0.808499	0.281887	0.327125	0.016*	
C21	0.47308 (11)	0.17198 (4)	0.45403 (8)	0.01312 (17)	
C22	0.36691 (11)	0.15892 (4)	0.35049 (8)	0.01327 (17)	
C23	0.29914 (11)	0.20820 (4)	0.28236 (8)	0.01541 (18)	
H23	0.229476	0.199464	0.212053	0.018*	
C24	0.33136 (12)	0.27053 (4)	0.31516 (8)	0.01597 (18)	
H24	0.284202	0.303707	0.267240	0.019*	
C25	0.43233 (11)	0.28369 (4)	0.41782 (8)	0.01448 (18)	
C26	0.50301 (11)	0.23437 (4)	0.48551 (8)	0.01430 (17)	
H26	0.573634	0.243537	0.555138	0.017*	
C111	0.86582 (11)	0.39220 (4)	0.40987 (8)	0.01383 (17)	
H111	0.784786	0.368751	0.456649	0.017*	
C121	1.16522 (14)	0.49466 (5)	0.14906 (9)	0.0233 (2)	
H12A	1.188699	0.536566	0.181970	0.035*	
H12B	1.105118	0.498388	0.069891	0.035*	
H12C	1.275896	0.472247	0.138464	0.035*	
C151	0.79088 (13)	0.17920 (4)	0.22231 (9)	0.01864 (19)	
H15A	0.769266	0.136629	0.192162	0.028*	
H15B	0.679003	0.200181	0.236846	0.028*	
H15C	0.858216	0.177225	0.298868	0.028*	
C211	0.55768 (11)	0.12487 (4)	0.53198 (8)	0.01470 (18)	
H211	0.632715	0.139016	0.595943	0.018*	
C221	0.24158 (15)	0.08339 (5)	0.21389 (9)	0.0238 (2)	
H22A	0.233090	0.037865	0.203679	0.036*	
H22B	0.302042	0.101530	0.143698	0.036*	
H22C	0.123897	0.101296	0.219223	0.036*	
C251	0.41248 (14)	0.39453 (4)	0.38939 (9)	0.0225 (2)	
H25A	0.453866	0.433561	0.426991	0.034*	
H25B	0.284384	0.394557	0.386171	0.034*	
H25C	0.458345	0.391238	0.306319	0.034*	

### 2,5-Dimethoxybenzaldehyde oxime (4) Atomic displacement parameters (Å^2^)

**Table:**

	*U*^11^	*U*^22^	*U*^33^	*U*^12^	*U*^13^	*U*^23^
O121	0.0203 (3)	0.0116 (3)	0.0174 (3)	−0.0029 (2)	0.0052 (2)	−0.0001 (2)
O221	0.0220 (3)	0.0133 (3)	0.0157 (3)	−0.0018 (2)	−0.0063 (2)	−0.0012 (2)
O151	0.0188 (3)	0.0128 (3)	0.0173 (3)	−0.0015 (2)	0.0015 (2)	−0.0033 (2)
O213	0.0230 (3)	0.0136 (3)	0.0189 (3)	0.0017 (3)	−0.0077 (3)	0.0021 (2)
O251	0.0208 (3)	0.0111 (3)	0.0223 (3)	−0.0006 (2)	−0.0048 (3)	0.0004 (2)
O113	0.0263 (4)	0.0160 (3)	0.0145 (3)	−0.0053 (3)	0.0077 (3)	−0.0043 (2)
N112	0.0186 (4)	0.0148 (4)	0.0129 (3)	−0.0001 (3)	0.0039 (3)	−0.0015 (3)
N212	0.0165 (4)	0.0146 (4)	0.0140 (4)	0.0020 (3)	−0.0027 (3)	0.0020 (3)
C11	0.0114 (4)	0.0128 (4)	0.0137 (4)	0.0010 (3)	−0.0013 (3)	−0.0004 (3)
C12	0.0123 (4)	0.0122 (4)	0.0158 (4)	0.0007 (3)	−0.0006 (3)	0.0003 (3)
C13	0.0151 (4)	0.0163 (4)	0.0142 (4)	0.0007 (3)	0.0015 (3)	0.0019 (3)
C14	0.0152 (4)	0.0173 (4)	0.0133 (4)	0.0021 (3)	0.0003 (3)	−0.0024 (3)
C15	0.0116 (4)	0.0126 (4)	0.0164 (4)	0.0013 (3)	−0.0030 (3)	−0.0015 (3)
C16	0.0117 (4)	0.0133 (4)	0.0146 (4)	0.0001 (3)	−0.0004 (3)	0.0010 (3)
C21	0.0125 (4)	0.0141 (4)	0.0128 (4)	−0.0005 (3)	0.0012 (3)	0.0007 (3)
C22	0.0126 (4)	0.0133 (4)	0.0139 (4)	−0.0012 (3)	0.0012 (3)	−0.0007 (3)
C23	0.0140 (4)	0.0175 (4)	0.0147 (4)	0.0001 (3)	−0.0017 (3)	−0.0001 (3)
C24	0.0145 (4)	0.0153 (4)	0.0180 (4)	0.0020 (3)	−0.0010 (3)	0.0026 (3)
C25	0.0128 (4)	0.0127 (4)	0.0179 (4)	−0.0009 (3)	0.0017 (3)	−0.0003 (3)
C26	0.0133 (4)	0.0159 (4)	0.0137 (4)	−0.0010 (3)	−0.0008 (3)	−0.0002 (3)
C111	0.0139 (4)	0.0135 (4)	0.0141 (4)	−0.0004 (3)	0.0016 (3)	0.0010 (3)
C121	0.0312 (5)	0.0160 (4)	0.0228 (5)	−0.0063 (4)	0.0096 (4)	0.0015 (4)
C151	0.0245 (5)	0.0131 (4)	0.0183 (4)	−0.0024 (3)	0.0002 (4)	−0.0005 (3)
C211	0.0145 (4)	0.0160 (4)	0.0135 (4)	−0.0011 (3)	−0.0018 (3)	−0.0005 (3)
C221	0.0321 (5)	0.0188 (4)	0.0204 (5)	−0.0043 (4)	−0.0119 (4)	−0.0020 (4)
C251	0.0280 (5)	0.0131 (4)	0.0263 (5)	0.0018 (4)	−0.0035 (4)	0.0027 (4)

### 2,5-Dimethoxybenzaldehyde oxime (4) Geometric parameters (Å, º)

**Table:**

O121—C12	1.3628 (10)	C21—C26	1.3935 (12)
O121—C121	1.4275 (11)	C21—C22	1.4151 (12)
O221—C22	1.3653 (10)	C21—C211	1.4651 (12)
O221—C221	1.4339 (11)	C22—C23	1.3866 (12)
O151—C15	1.3721 (10)	C23—C24	1.3988 (12)
O151—C151	1.4291 (11)	C23—H23	0.9500
O213—N212	1.4029 (9)	C24—C25	1.3858 (12)
O213—H213	0.877 (15)	C24—H24	0.9500
O251—C25	1.3706 (10)	C25—C26	1.3929 (12)
O251—C251	1.4268 (11)	C26—H26	0.9500
O113—N112	1.4017 (9)	C111—H111	0.9500
O113—H113	0.875 (16)	C121—H12A	0.9800
N112—C111	1.2747 (11)	C121—H12B	0.9800
N212—C211	1.2767 (12)	C121—H12C	0.9800
C11—C16	1.4020 (12)	C151—H15A	0.9800
C11—C12	1.4072 (12)	C151—H15B	0.9800
C11—C111	1.4639 (12)	C151—H15C	0.9800
C12—C13	1.3962 (12)	C211—H211	0.9500
C13—C14	1.3908 (12)	C221—H22A	0.9800
C13—H13	0.9500	C221—H22B	0.9800
C14—C15	1.3921 (12)	C221—H22C	0.9800
C14—H14	0.9500	C251—H25A	0.9800
C15—C16	1.3887 (12)	C251—H25B	0.9800
C16—H16	0.9500	C251—H25C	0.9800
			
C12—O121—C121	118.13 (7)	C23—C24—H24	120.1
C22—O221—C221	117.40 (7)	O251—C25—C24	125.45 (8)
C15—O151—C151	116.42 (7)	O251—C25—C26	115.32 (8)
N212—O213—H213	101.5 (10)	C24—C25—C26	119.23 (8)
C25—O251—C251	117.38 (7)	C25—C26—C21	121.89 (8)
N112—O113—H113	100.6 (10)	C25—C26—H26	119.1
C111—N112—O113	111.63 (7)	C21—C26—H26	119.1
C211—N212—O213	111.12 (7)	N112—C111—C11	123.34 (8)
C16—C11—C12	119.24 (8)	N112—C111—H111	118.3
C16—C11—C111	115.96 (8)	C11—C111—H111	118.3
C12—C11—C111	124.79 (8)	O121—C121—H12A	109.5
O121—C12—C13	123.99 (8)	O121—C121—H12B	109.5
O121—C12—C11	116.60 (8)	H12A—C121—H12B	109.5
C13—C12—C11	119.41 (8)	O121—C121—H12C	109.5
C14—C13—C12	120.53 (8)	H12A—C121—H12C	109.5
C14—C13—H13	119.7	H12B—C121—H12C	109.5
C12—C13—H13	119.7	O151—C151—H15A	109.5
C13—C14—C15	120.42 (8)	O151—C151—H15B	109.5
C13—C14—H14	119.8	H15A—C151—H15B	109.5
C15—C14—H14	119.8	O151—C151—H15C	109.5
O151—C15—C16	124.23 (8)	H15A—C151—H15C	109.5
O151—C15—C14	116.36 (8)	H15B—C151—H15C	109.5
C16—C15—C14	119.40 (8)	N212—C211—C21	123.80 (8)
C15—C16—C11	120.99 (8)	N212—C211—H211	118.1
C15—C16—H16	119.5	C21—C211—H211	118.1
C11—C16—H16	119.5	O221—C221—H22A	109.5
C26—C21—C22	118.53 (8)	O221—C221—H22B	109.5
C26—C21—C211	116.18 (8)	H22A—C221—H22B	109.5
C22—C21—C211	125.29 (8)	O221—C221—H22C	109.5
O221—C22—C23	123.76 (8)	H22A—C221—H22C	109.5
O221—C22—C21	116.92 (8)	H22B—C221—H22C	109.5
C23—C22—C21	119.32 (8)	O251—C251—H25A	109.5
C22—C23—C24	121.27 (8)	O251—C251—H25B	109.5
C22—C23—H23	119.4	H25A—C251—H25B	109.5
C24—C23—H23	119.4	O251—C251—H25C	109.5
C25—C24—C23	119.74 (8)	H25A—C251—H25C	109.5
C25—C24—H24	120.1	H25B—C251—H25C	109.5
			
C121—O121—C12—C13	−4.21 (13)	C211—C21—C22—O221	1.91 (13)
C121—O121—C12—C11	175.24 (8)	C26—C21—C22—C23	1.31 (12)
C16—C11—C12—O121	−179.74 (7)	C211—C21—C22—C23	−177.99 (8)
C111—C11—C12—O121	−0.50 (13)	O221—C22—C23—C24	179.06 (8)
C16—C11—C12—C13	−0.26 (13)	C21—C22—C23—C24	−1.05 (13)
C111—C11—C12—C13	178.98 (8)	C22—C23—C24—C25	−0.22 (13)
O121—C12—C13—C14	178.77 (8)	C251—O251—C25—C24	−3.63 (13)
C11—C12—C13—C14	−0.67 (13)	C251—O251—C25—C26	175.86 (8)
C12—C13—C14—C15	0.80 (13)	C23—C24—C25—O251	−179.32 (8)
C151—O151—C15—C16	0.74 (12)	C23—C24—C25—C26	1.21 (13)
C151—O151—C15—C14	179.85 (8)	O251—C25—C26—C21	179.53 (8)
C13—C14—C15—O151	−179.15 (8)	C24—C25—C26—C21	−0.94 (13)
C13—C14—C15—C16	0.01 (13)	C22—C21—C26—C25	−0.32 (13)
O151—C15—C16—C11	178.13 (8)	C211—C21—C26—C25	179.03 (8)
C14—C15—C16—C11	−0.95 (13)	O113—N112—C111—C11	−179.60 (8)
C12—C11—C16—C15	1.08 (13)	C16—C11—C111—N112	168.18 (8)
C111—C11—C16—C15	−178.23 (8)	C12—C11—C111—N112	−11.08 (14)
C221—O221—C22—C23	4.54 (13)	O213—N212—C211—C21	−178.99 (8)
C221—O221—C22—C21	−175.35 (8)	C26—C21—C211—N212	176.46 (8)
C26—C21—C22—O221	−178.79 (8)	C22—C21—C211—N212	−4.24 (14)

### 2,5-Dimethoxybenzaldehyde oxime (4) Hydrogen-bond geometry (Å, º)

Cg1 and Cg2 are the centroids of the C11–C16 and C21–C26 rings, respectively.

**Table:**

*D*—H···*A*	*D*—H	H···*A*	*D*···*A*	*D*—H···*A*
O113—H113···O121^i^	0.875 (16)	2.247 (15)	2.8944 (9)	130.7 (12)
O113—H113···N112^i^	0.875 (16)	1.965 (16)	2.7567 (10)	149.9 (13)
O213—H213···O221^ii^	0.877 (15)	2.204 (15)	2.8758 (9)	133.1 (12)
O213—H213···N212^ii^	0.877 (15)	2.034 (15)	2.8160 (10)	147.9 (13)
C111—H111···O251	0.95	2.46	3.2458 (11)	140
C121—H12*C*···N212^iii^	0.98	2.53	3.4400 (13)	155
C151—H15*A*···O113^iv^	0.98	2.50	3.3947 (11)	152
C14—H14···*Cg*2^iii^	0.95	2.98	3.6656 (9)	130
C151—H15*B*···*Cg*2	0.98	2.72	3.5973 (10)	149
C24—H24···*Cg*1^v^	0.95	2.67	3.4281 (10)	137
C211—H211···*Cg*1^vi^	0.95	2.78	3.6272 (9)	149

Symmetry codes: (i) −*x*+2, −*y*+1, −*z*+1; (ii) −*x*+1, −*y*, −*z*+1; (iii) *x*+1, −*y*+1/2, *z*−1/2; (iv) *x*, −*y*+1/2, *z*−1/2; (v) *x*−1, *y*, *z*; (vi) *x*, −*y*+1/2, *z*+1/2.
